# Laparoscopic intraarterial catheterization with selective ICG fluorescence imaging in colorectal surgery

**DOI:** 10.1038/s41598-021-94244-y

**Published:** 2021-07-20

**Authors:** Christian Heiliger, Jerzy Piecuch, Alexander Frank, Dorian Andrade, Viktor von Ehrlich-Treuenstätt, Dobromira Evtimova, Florian Kühn, Jens Werner, Konrad Karcz

**Affiliations:** 1 Department of General, Visceral, and Transplantation Surgery, Hospital of the LMU Munich, Ludwig-Maximilians-University (LMU), Marchioninistrasse 15, 81377 Munich, Germany; 2 Klinika Chirurgii Ogolnej, Metabolicznej i Medycyny Ratunkowej w Zabrzu, Slaski Universytet Medyczny w Katowicach, Katowicach, Poland

**Keywords:** Medical research, Preclinical research, Medical imaging, Rectum

## Abstract

The quality of mesorectal resection is crucial for resection in rectal cancer, which should be performed by laparoscopy for better outcome. The use of indocyanine green (ICG) fluorescence is now routinely used in some centers to evaluate bowel perfusion. Previous studies have demonstrated in animal models that selective intra-arterial ICG staining can be used to define and visualize resection margins in rectal cancer. In this animal study, we investigate if laparoscopic intra-arterial catheterization is feasible and the staining of resection margins when performing total mesorectal excision with a laparoscopic medial to lateral approach is possible. In 4 pigs, laparoscopic catheterization of the inferior mesenteric artery (IMA) is performed using a seldinger technique. After a bolus injection of 10 ml ICG with a concentration of 0.25 mg/ml, a continuous intra-arterial perfusion was established at a rate of 2 ml/min. The quality of the staining was evaluated qualitatively. Laparoscopic catheterization was possible in all cases, and the average time for this was 30.25 ± 3.54 min. We observed a significant fluorescent signal in all areas of the IMA supplied, but not in other parts of the abdominal cavity or organs. In addition, the mesorectum showed a sharp border between stained and unstained tissue. Intraoperative isolated fluorescence augmentation of the rectum, including the mesorectum by laparoscopic catheterization, is feasible. Inferior mesenteric artery catheterization and ICG perfusion can provide a fluorescence-guided roadmap to identify the correct plane in total mesorectal excision, which should be investigated in further studies.

## Introduction

Intraarterial Indocyanine Green (ICG) perfusion into the main tumor supplying artery may help to identify the correct resection borders in oncological surgery. As described before, a continuous ICG-perfusion via IMA (inferior mesenteric artery) may visualize the correct plane for total mesorectal excision (TME) in the case of rectal cancer^1–3^. Forgione et al. were able to confirm our concept^3^. This technique may improve the quality and safety of mesorectal resection, a faster learning curve and improve overall neurological outcome.

It is known that the quality of mesorectal resection is crucial for predicting the local recurrence risk^4^. Patients benefit from minimal-invasive rectal excision due to short-term recovery, fewer surgical site infections, and less overall blood loss^5^ while the longterm oncological outcome seems to be comparable in the open versus laparoscopic approach^4,6^. This suggests that the laparoscopic approach for lower rectal excision and TME might be the gold standard for surgery of rectal cancer. However, Creavin et al. showed that laparoscopic resections were associated with a higher rate of incomplete mesorectal excisions^4^. As mentioned before, this implicates a higher risk for local recurrence. The reason might belong learning curves for the technique involved, depending on the individual skill of the surgeon and also higher conversion rates in less trained surgeons^7,8^.

In the last few years, fluorescence-guided surgery (FGS) with ICG has become widespread in several medical and surgical specialties^9^. There are also few studies regarding laparoscopic liver surgery, examining if intraarterial ICG-injection can help identify resection borders. In these studies, a hybrid operative suite with a transfemoral approach is used to catheterize the arteries^10,11^. Forgione et al. were able to show that staining of the mesocolon via a transfemoral approach is also possible^3^.

One limitation occurring in our previous study was the need for laparotomy for puncturing the IMA^1^. As described above, interventional catheterization would be an option, but just a few hospitals have access to a hybrid operative suite and the risk of complications is described between 0.05 and 2%^12,13^.

Another option would be laparoscopic artery catheterization. This is described in the literature, mainly for hepatic artery catheterization for regional chemotherapy^14–16^.

In this animal study, we want to examine if a laparoscopic approach with laparoscopic catheterization and ICG-perfusion of the IMA and image-guided identification of the resection areas of the mesorectum with near-infrared angiographic enhancement is possible and feasible.

## Material and methods

### Animals

A total of two swine (weight: 40–45 kg, age: 3–4 months old) were used in this non-survival study. All procedures were carried out in strict accordance with recommendations and guidance for the care and use of laboratory animals of the National Institutes of Health, which received full approval by the local Ethical Committee on Animal Experimentation (FIM-18105) by Fördergemeinschaft für Innovative Medizin in Beichlingen, Germany and Ludwig-Maximilians-University (LMU). The study was carried out in compliance with the ARRIVE guideline. Induction was achieved using Azaperon (Stresnil), i.m. (2 mg/kg) combined with Atropine s.c. (0.02–0.1 mg/kg) and Ketamin 10% i.m. (20 mg/kg). Anesthesia was maintained with 2% Isoflurane, Pancuronium, Meloxicam, Metamizole, Ketamine, and, if necessary, Fentanyl. At the end of the procedure, animals were humanely killed with an intravenous injection of a lethal dose of T61.

### Surgical procedure and laparoscopic ICG video angiography

The surgical approach was achieved in the following fashion: The IMA was prepared with a medial-to-lateral approach after standard laparoscopy. For temporary closure, we used a small bulldog clamp, which was put into the abdomen through a 12 mm trocar, and applied it to the distal IMA. The first step during the medial approach for rectum resection is ligating the vessel of the inferior mesenteric artery. We did this by a proximal clipping technique followed by the final cutting of the IMA (see Supplementary Video^17^ [00:06–01:12]).

Subsequently, we inserted an introducer system (Arrow, Two-Lumen Central Venous Catheterization Set, Teleflex Medical GmbH Willy-Rüsch-Str. 4–10 D-71394 Kernen) in Seldinger technique and applied the catheter (Pajunk, SonoLong Echo NanoLine, Pajunk GmbH Medizintechnologie Karl-Hall-Strasse 1 78187 Geisingen Germany). After the arteriae section, we placed the catheter into the IMA and fixed it with two ligatures preventing retrograde flow. After the bulldog clamp was removed, we checked for bleeding (see Supplementary Video^17^ [01:15–02:01]).

As soon as locating the rectum and mesorectum was achieved, ICG was applied to the IMA. Therefore, we established continuous intra-arterial perfusion via a syringe pump of ICG. ICG (Pulsion Medical Systems SE, Hans-Riedl-Str. 21, 85622 Feldkirchen, Germany) concentration was 0.25 mg/ml mixed with aqua ad iniectabilia with a perfusion rate of 2 ml/min. We started with a bolus of 10 ml. Fluorescence-imaging was observed with a NIR camera by real-time overlapping to the real camera image (Maxer Viron X, Maxer Medizintechnik GmbH, 78549 Spaichingen, Germany). Finally, we started mesorectal excision in standard surgical fashion (see Supplementary Video^17^ [02:08–05:20]).

The time for laproscopic catheterization was defined from identification of the IMA to completion of the last ligation. All data are presented as mean ± standard deviation (SD).

## Results

**Figure 1:**
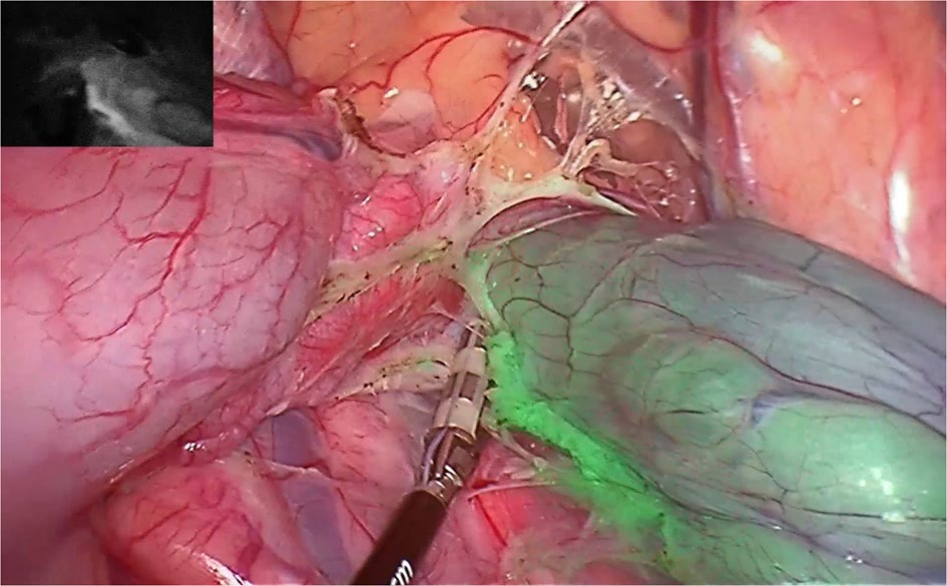
Laparoscopic view into the small pelvis from the left side during resection with an overlay image of the ICG signal. The rectum and mesorectum show a robust infrared signal, as you can see on the little black and white picture in the left top corner.

**Figure 2:**
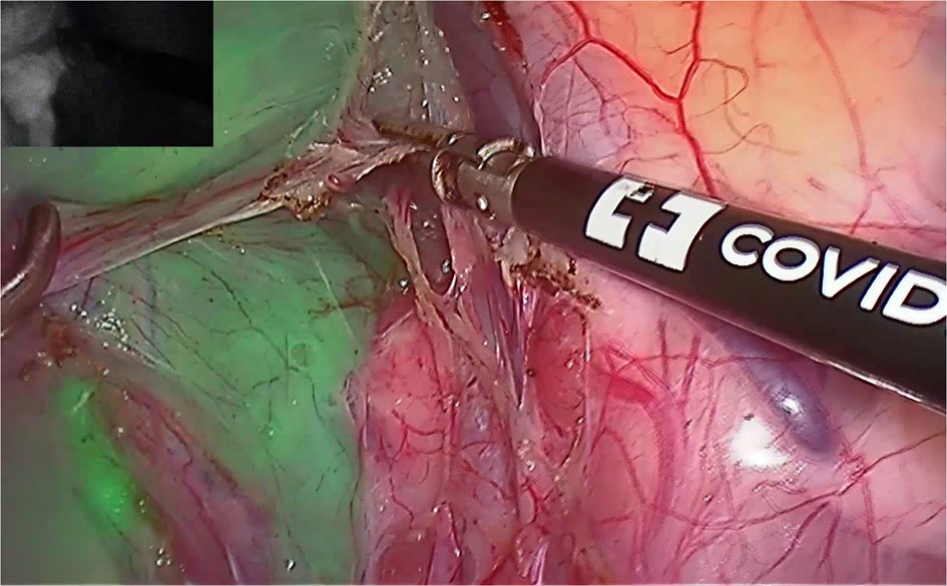
Laparoscopic view into the small pelvis from the right side during total mesorectal excision and dissection of the planes.

**Figure 3:**
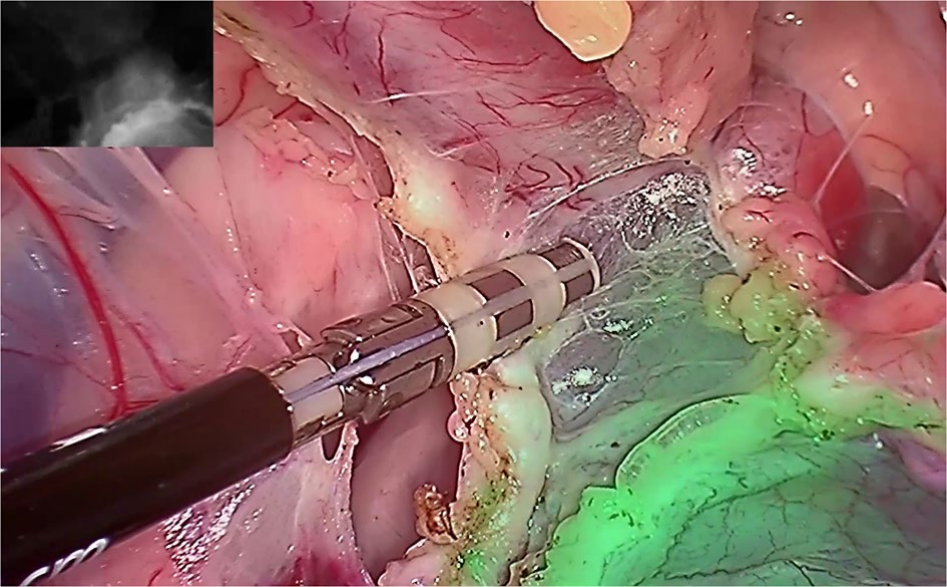
Laparoscopic view into the small pelvis from the right side during total mesorectal excision deep in the pelvis. Notice how the ventral part of the pelvis is not colored, and the ICG signal is also shown at the very bottom of the rectum.

After an initial bolus of 10 ml of ICG (this is a total of 2.5 mg ICG), we directly observed an infrared signal. The overlay video-image showed a promising ICG-perfusion of the rectum and mesorectum without an infrared signal of other parts of the situs. We observed a sharp border between colored and non-colored tissue, and this border seemed to match the visceral fasciae (Figs. 1, 2, 3). We stopped continuous perfusion after 8 min (total of 6.5 mg ICG in 26 ml) because of an optically sufficient infrared signal of the rectum and mesorectum. Furthermore, we wanted to prevent further systemic ICG application as ICG accumulation in the liver was already noticeable. The time for laparoscopic catheterization was 30.25 ± 3.54 min.

During mesorectal resection, which we mainly performed with a Ligasure Device (Ligasure, Medtronic GmbH, Earl-Bakken-Platz 1, 40670 Meerbusch), you can see how visceral and parietal fasciae even under blunt dissection separate and how infrared signals match (Fig. 2). While sealing and cutting adhesions some ICG remains on the instrument, so we cleaned the instrument a few times during surgery to prevent ICG contamination of other parts of the situs. The ICG signal was observed until the very end of the rectum at the bottom of the wall of the pelvis (Fig. 3, see Supplementary Video^17^ [02:08–05:20]). After 40 min without further ICGperfusion, the ICG signal did not change subjectively.

## Discussion

The quality of mesorectal excision is mostly related to complete resection of the fascial envelope, and as mentioned before, the quality of mesorectal resection is crucial for predicting local recurrence risk^4^. This image-guided approach may help the surgeon identify the correct plane faster and safer. In our experience, most surgeons favor a laparoscopic approach for rectal resection, if possible. For a medial-to-lateral approach, laparoscopic intraarterial catheterization would not even change operative workflow.

Minimally invasive catheter placement for regional chemotherapy into the hepatic artery using conventional laparoscopic techniques has been described in literature^15,16,18^. Franklin et al. reported a series of 20 catheterizations without intraoperative complications^16^. Van Nieuwenhove et al. reported a series of 29 catheterizations without major perioperative complications^15^. Cheng et al. reported a case series of 38 patients with laparoscopic hepatic artery infusion pump (LHAIP) implantations without major intraoperative complications^18^. All authors describe laparoscopic catheterization as a challenging but safe and feasible procedure^15,16,18^, which accords with our experience. In our approach the catheterized vessel.

(IMA) will be resected. Postoperative complications should not occur relating to catheterization. Furthermore, the dissection of the IMA is way more straightforward than the dissection of hepatic vessels. There are also case reports in literature regarding catheterization of arteries with robotic systems such as the DaVinci, which may decrease the time for catheterization and improve safeness^19^.

With a mean time of 30.25 ± 3.54 min for catheterization, our approach is comparable to interventional catheterization described by Forgione et al. but without the risks of additional intervention^3^.

Nevertheless, even though dissecting the artery and the catheterization technique might be timeconsuming in the beginning, the new technique holds great potential to be time-saving procedure-time during mesorectal excision because of better and faster identification of the correct resection plane.

In previous experiments^1,2^, a rapid ICG washout was observed with intra-arterial administration, so the experiments were performed with continuous administration. Forgione et al. have investigated this both quantitatively and qualitatively and have come to the results here of an optimal infusion rate of 20 ml/h with a dosage of 0.01 mg/kg^3^. This is roughly in line with our dosage of 6.5 mg in total. Continuous perfusion appears to be an important factor for adequate visualization of the mesorectum, presumably this will also be a decisive factor in humans, although the exact length of the catheter and appropriate catheter openings may also play a major role here.

Another possibility that is created by arterial catheterization is regional chemotherapy. Even if controversial, some studies suggest that regional chemotherapy may improve outcomes in some patient groups^20^. Also, new surgical methods like TaTME could improve from this technique. Identification of the embryonal planes during transanal preparation maybe improve correct dissection along embryonal planes.

In our opinion, this technique may offer a new approach in oncological surgery and may, especially in rectal surgery, improve the outcome. At the end: further studies must deal with some crucial questions:

Which application method works best and offers the best integration into the standard operating procedures: Laparoscopic or robotic catheterization in comparison to interventional catheterization with a hybrid operating room or preoperative catheterization by interventional radiologists.

Which Dose, Volume, and perfusion-rate of ICG offer the best results?

Does the ICG-perfused area correspond to the real fascia conditions in humans?

Is the additional effort worth it? Does this new approach improve circumferential resection margin, locoregional recurrence-free survival, urogenital dysfunction, the learning curve, and duration of the surgery?

## Conclusion

Intraoperative isolated fluorescence augmentation of the rectum, including the mesorectum by laparoscopic catheterization, is feasible. By catheterization of the inferior mesenteric artery and ICG-perfusion, you may achieve a fluorescence-guided roadmap to identify the right plane during total mesorectal excision, which should be examined in further studies.

## Supplementary Information

Supplementary Information.
