# Supplementary material for: Laparoscopic intraarterial catheterization with selective ICG fluorescence imaging in colorectal surgery
Source: Sci Rep. 2021 Jul 20;11:14753. doi: 10.1038/s41598-021-94244-y (PMC8292501; doi:10.1038/s41598-021-94244-y)
Supplement: Supplementary file 1 — Supplementary Information. [file 41598_2021_94244_MOESM1_ESM.docx]

**Laparoscopic intraarterial catheterization with selective ICG fluorescence imaging in colorectal surgery**

**VIDEO**

<https://doi.org/10.6084/m9.figshare.11955165.v1>

Christian Heiliger^1^, PD Dr. Jerzy Piecuch^2^, Dr. Alexander Frank^1^, Dorian Andrade^1^, Dr. Viktor von Ehrlich-Treuenstätt^1^, Dr. Dobromira Evtimova^1^, PD Dr. Florian Kühn^1^, Prof. Dr. Jens Werner^1^, Prof. Dr. Konrad Karcz^1^

1. *Ludwig-Maximilians-University (LMU), Department of General, Visceral, and Transplantation Surgery, Hospital of the LMU Munich, Munich 81377, Germany*
2. *Klinika Chirurgii Ogolnej, Metabolicznej i Medycyny Ratunkowej w Zabrzu, Slaski Universytet Medyczny w Katowicach, Poland*

**Background**

The quality of mesorectal resection is crucial for resection in rectal cancer, which should be performed by laparoscopy for better outcome. The use of indocyanine green (ICG)fluorescence is now routinely used in some centers to evaluate bowel perfusion. Previous studies have demonstrated in animal models that selective intra-arterial ICG staining can be used to define and visualize resection margins in rectal cancer. In this animal study, we investigate if laparoscopic intra-arterial catheterization is feasible and the staining of resection margins when performing total mesorectal excision (TME) with a laparoscopic medial to lateral approach is possible.

**Methods**

In 4 pigs, laparoscopic catheterization of the inferior mesenteric artery (IMA) is performed using a seldinger technique. After a bolus injection of 10 ml ICG with a concentration of 0.25 mg/ml, a continuous intra-arterial perfusion was established at a rate of 2 ml/min. The quality of the staining was evaluated qualitatively.

**Results**

Laparoscopic catheterization was possible in all cases, and the average time for this was 30,25±3,54 min. [CH1] We observed a significant fluorescent signal in all areas of the IMA supplied, but not in other parts of the abdominal cavity or organs. In addition, the mesorectum showed a sharp border between stained and unstained tissue.

**Conclusion**

Intraoperative isolated fluorescence augmentation of the rectum, including the mesorectum by laparoscopic catheterization, is feasible. Inferior mesenteric artery catheterization and ICG perfusion can provide a fluorescence-guided roadmap to identify the correct plane in total mesorectal excision, which should be investigated in further studies.
